# P19 H-Ras Induces G1/S Phase Delay Maintaining Cells in a Reversible Quiescence State

**DOI:** 10.1371/journal.pone.0008513

**Published:** 2009-12-30

**Authors:** Maria Camats, Mariette Kokolo, Kate J. Heesom, Michael Ladomery, Montse Bach-Elias

**Affiliations:** 1 Unidad de Splicing, Instituto de Investigaciones Biomédicas de Barcelona-Consejo Superior de Investigaciones Científicas, Barcelona, Spain; 2 University of Bristol Proteomics Facility, Bristol, United Kingdom; 3 Centre for Research in Biomedicine, Faculty of Health and Life Sciences, University of the West of England, Bristol, United Kingdom; Roswell Park Cancer Institute, United States of America

## Abstract

**Background:**

Three functional c-*ras* genes, known as c-H-*ras*, c-K-*ras*, and c-N-*ras*, have been largely studied in mammalian cells with important insights into normal and tumorigenic cellular signal transduction events. Two K-Ras mRNAs are obtained from the same pre-mRNA by alternative splicing. H-Ras pre-mRNA can also be alternatively spliced in the IDX and 4A terminal exons, yielding the p19 and p21 proteins, respectively. However, despite the Ras gene family's established role in tumorigenic cellular signal transduction events, little is known about p19 function. Previous results showed that p19 did not interact with two known p21 effectors, Raf1 and Rin1, but was shown to interact with RACK1, a scaffolding protein that promotes multi-protein complexes in different signaling pathways (Cancer Res 2003, 63 p5178). This observation suggests that p19 and p21 play differential and complementary roles in the cell.

**Principal Findings:**

We found that p19 regulates telomerase activity through its interaction with p73α/β proteins. We also found that p19 overexpression induces G1/S phase delay; an observation that correlates with hypophosphorylation of both Akt and p70SK6. Similarly, we also observed that FOXO1 is upregulated when p19 is overexpressed. The three observations of (1) hypophosphorylation of Akt, (2) G1/S phase delay and (3) upregulation of FOXO1 lead us to conclude that p19 induces G1/S phase delay, thereby maintaining cells in a reversible quiescence state and preventing entry into apoptosis. We then assessed the effect of p19 RNAi on HeLa cell growth and found that p19 RNAi increases cell growth, thereby having the opposite effect of arrest of the G1/S phase or producing a cellular quiescence state.

**Significance:**

Interestingly, p19 induces FOXO1 that in combination with the G1/S phase delay and hypophosphorylation of both Akt and p70SK6 leads to maintenance of a reversible cellular quiescence state, thereby preventing entry into apoptosis.

## Introduction

Three functional c-*ras* genes, known as c-H-*ras*, c-K-*ras*, and c-N-*ras*, have been largely studied in mammalian cells with important insights into normal and tumorigenic cellular signal transduction events [1], [2], [3]. In c-H-*ras*, c-K-*ras*, and c-N-*ras*, codons 12 and 61 are ‘hotspots’ for mutations that activate their malignant transforming properties [1], [2], [3]. H-*ras* mutations were also shown to be an important hallmark in Costello syndrome, a rare multi-system disease that affects major organs [4], [5]. Additionally, H*-ras* knockout (KO) mice were found to be viable, indicating that H-*ras* is dispensable for embryogenesis [6]. Two K-Ras mRNAs are obtained from the same pre-mRNA by alternative splicing [7], [8]. H-Ras pre-mRNA can also be alternatively spliced in the IDX and 4A terminal exons, yielding the p19 and p21 proteins, respectively [9], [10], [11]. However, despite the Ras gene family's established role in tumorigenic cellular signal transduction events, little is known about p19 function.

To date, p19 is known to be conserved in all mammalian cells and to localize in the nucleus/cytosol [11]. Interestingly, unlike p21, p19 does not bind to GTP [11], thereby indicating that these Ras proteins have distinct functions. In addition, p19 was demonstrated to bind RACK1 [11], a scaffolding protein that brings together different factors, enabling them to act in a common pathway, e.g. mitogen-activated protein kinase pathways [12], [13]. RACK1 also binds and activates PKCβII and SRC [12], [13]. Finally, interaction between p19 and p73β was shown to abolish the MDM2-mediated transcriptional repression of p73β [14].

Tuberous sclerosis complex (TSC) is an autosomal disease associated with the formation of usually benign tumors caused by mutations in either the *TSC1* or *TSC2* tumor suppressor genes that are known to be involved in the TSC pathway [15]. Rheb, a GTPase, plays a central role in the TSC pathway [15] in which it is a direct target of TSC2 and is negatively regulated by the GTPase-activating protein (GAP) activity of TSC2. Recently, TCTP, a highly conserved protein upregulated in various tumors, has been shown to be an essential factor for growth and proliferation, and to have guanosine exchange factor (GEF) activity for Rheb [16]. The TSC pathway is also known to be regulated by Akt and ERK phosphorylation [15].

In our study, we show that overexpression of p19 regulates TCTP levels as well as Akt and ERK phosphorylation, that finally leads to inactivation of p70S6K. Additionally, p19 was found to activate telomerase activity through p73 and arrest cells at the G1/S phase, i.e. in a reversible cellular quiescence state, thereby preventing entry into apoptosis.

## Results

### P19 Activates PKC and the ERK1 Pathway

**Figure 1:**
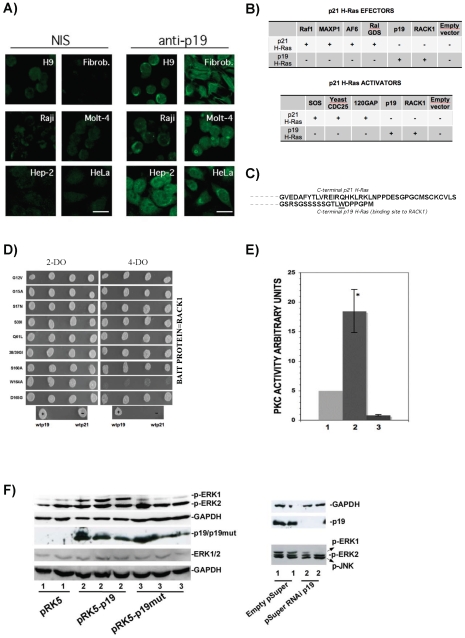
P19 activates PKC and ERK1. A) Subcellular localization of endogenous p19 in different human cell types detected by indirect immunofluoresence (IF) with antibody SP57 (1/500). Cells used are H9, fibroblasts (Fibrob.), Raji, Molt-4, Hep-2, and HeLa. Secondary antibody was fluorescein-labeled (FITC) anti-rabbit (1/3000). Bar = 20 µm. Corresponding control images performed with non-immune serum of SP57 are shown in the Figure as NIS. Yeast two-hybrid assay on 4-DO (-Leu/-Trp/-His/-Ade) as compared to 2-DO (-Leu/-Trp) plates to assess the binding potential of p19 to p21 effectors Raf1, MAXP1, AF6, and RalGDS compared to p21. P19 and p21 were cloned in the pGBKT7 vector and Raf1, MAXP1, AF6, RalGDS, SOS, Yeast CDC25 and 120GAP in the pGADT7 vector. As positive controls, the previously described bindings between p19-p19 and p19-RACK1 [11] were also presented. A plus sign (+) indicates interaction. C) Amino acid sequences of the C-terminal of p21 H-Ras (upper) and p19 H-Ras (lower). Position 164(W), is underlined. D) Yeast two-hybrid assay of the binding potential of p19 mutants (G12V, G15A, S17N, S39I, Q61L; 38/39GI, S160A, W164A, D165G) with the RACK1 bait protein grown on 2-DO and 4-DO yeast-selective plates co-expressing the pGBKT7-p19 mutants and pGADT7-RACK1. Lower four panels are positive and negative controls: (+) wild-type p19 x RACK1 on 2-DO (left) and 4-DO (right) and (−) wild-type p21 x RACK1 on 2-DO (left) and 4-DO (right). E) PKC activity assay in HeLa cells transiently overexpressing (1) empty vector, (2) p19, and (3) p19mut. PKC activity is given under arbitrary units. The value of HeLa cells overexpressing empty vector was set to 5 and considered as basal PKC activity (*P<0.001). F) On the left, western blot analysis showing the activation of ERK1 by detecting p-ERK1 (phosphorylated-ERK1). Lanes 1, control cells; empty vector (basal levels); lanes 2, p19; lanes 3, p19mut. Upper panel, anti-p-ERK1/2 (p-T202/Y204); second panel, anti-GAPDH (internal unchanged control); third panel, overexpression of p19 (lanes 2) or p19mut (lanes 3). A 3-fold increase of p-ERK1 were in lanes 2 compared to lanes 1. Unchanged total ERK1/2 was also found (lower panels) compared to the corresponding GAPDH internal control. On the right, p 19 RNAi is shown (upper gels) and was seen to reduce p-ERK1 levels (lower panel), but not p-ERK2 and p-JNK levels.

Previously, p19 was suggested to interact with RACK1 and co-localize in the perinuclear region [11]. Using the Fluorescence Resonance Energy Transfer (FRET) technique, we found a 15% FRET efficiency between ECFP-RACK1 (donor) and EYFP-p19 (acceptor) at the perinuclear region, thereby confirming the localization of p19 (our results not shown). Moreover, the perinuclear region and nucleus were also mainly found to be decorated with anti-p19 antibody SP57 in different human cells type (see Figure 1A). Additionally, to further underscore the different role of p19 with respect to the p21 isoform, p19 showed no protein-binding activity to not only the p21 effectors Raf1, MAXP1, AF6, and Ral-GDS (Figure 1B), but also the p21 activators SOS, yeast CDC25, and p120GAP (Figure 1B). These findings are in line with that fact that p19 does not localize to the plasma membrane (Figure 1A). They also indicate that p19 does not interfere with p21 activity by sequestering or downmodulating these effectors and activators by direct protein-protein contact. We have not compared p21 and p19 in all the experiments here presented, because: i) as p19 has been detected in different compartments (Figure 1A) and ii) as there is no binding of p19 to p21 effectors/activators (Figure 1B), we deduce then a different function for p19. Furthermore, p21 has been hardly studied and we believed, then, it is not necessary to repeat again taking into account that, in our hands, p21 even does not express at the same level as p19.

A distinguishing feature of p19 compared to p21 is its C-terminal amino acid sequence GSRSGSSSSSGTLWDPPGPM (see Figure 1C) that contains the binding site to RACK1 [11]. To further analyze p19-RACK1 interaction, the C-terminal of p19 was mutated at three positions, S160A, W164A, and D165G, and each single mutation was analyzed by yeast two-hybrid assays. Several mutations of p21 were described to produce a transforming phenotype of the *H-ras* gene [1], [2], [3]. Codons 12 and 61 are ‘hotspots’ for mutations that activate their malignant transforming properties. Mutations on codon 38 and 39 affect the effector domain of p21 and S17N is a dominant negative H-ras mutant. Since these mutations are localized in the common region between p19 and p21, we also investigated the effect of these mutations on p19-RACK1 binding using yeast two-hybrid assays. As shown in Figure 1D of all the mutations tested, only the W164A mutant prevented p19-RACK1 binding. The inhibition of the RACK1-p19 interaction is not due to a mis-targeting to different compartment, since we detected the same p19 and RACK1 perinuclear staining when the W164A mutant was overexpressed (our unpublished results). RACK1 is a scaffolding protein that assemblies multimeric protein complexes, several of which contain PKCβII [17]. As RACK1 and PKCβII have been detected to co-localize at the perinuclear region [17], the co-localization of p19 and PKCβII was analyzed and showed to also co-localize at the perinuclear region (Figure S1). This finding adds further credence to our hypothesis that p19, RACK1, and PKCβII may form a multimeric protein-complex with a conjointly action at the perinuclear region, being this possible thus RACK1 and PKCβII have specific protein-protein binding sites distinct to the RACK1-p19 binding site. Consequently, we analyzed the effect of p19 on PKC activity using overexpressed p19 in HeLa cells. We found a 3 to 4.5-fold increase of PKC activity compared to cells expressing empty vector or mutant p19W164A (p19mut) (Figure 1E), thereby showing that p19 strongly induces PKC activity.

The upregulation of PKC by p19 prompted us to analyze the phosphorylation levels of the mitogen-activated protein kinases ERK1 and 2, p38 MAPKα, β and γ, JNK1, 2 and 3, and the transcriptional activator p-Jun in HeLa cells overexpressing either p19 or p19mut. Of the nine factors tested, p19 only induced a change of phosphorylation level in ERK1 (Figure 1F and unpublished results). Moreover, the RNAi of p19 was also found to reduce the level of p-ERK1 without affecting p-ERK2 or p-JNK (Figure 1F). As PKC may also activate the ERK-pathway independently of external growth factors [18], the activation of p-ERK1 by p19 (Figure 1F) correlates with the PKC activity results (Figure 1E).

### P19 Induces the G1/S Delay and Inactivates SRC

**Figure 2:**
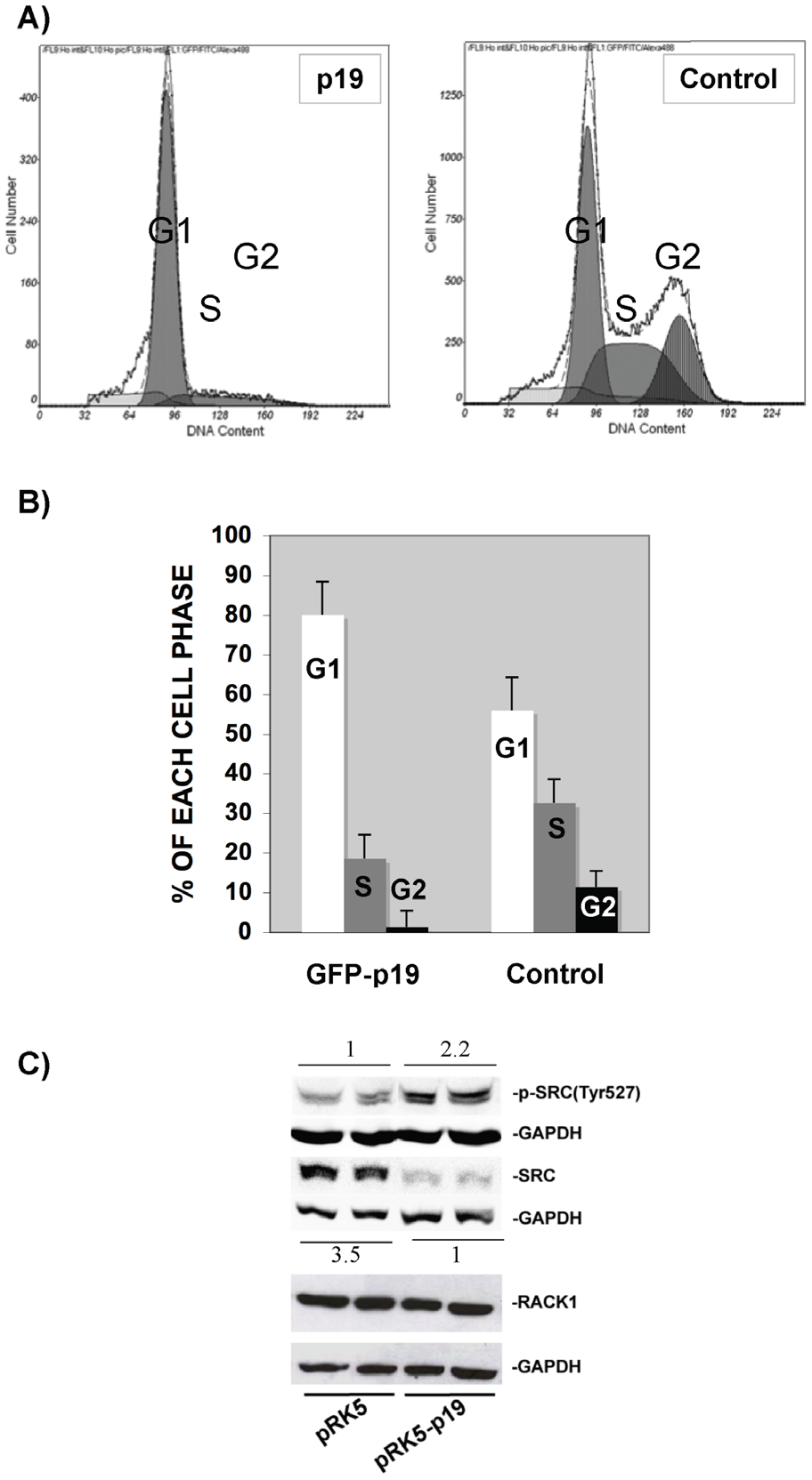
Increasing levels of p19 induces G1/S cell phase delay and SRC inactivation. A) Cell cycle phase analysis by FACS (fluorescent-activated cell sorting) in HeLa cells overexpressing GFP-p19 or GFP only (negative control). A representative flow cytometry histogram is shown. B). G1, S, and G2 percentages are drawn on the Figure. G1 columns *P* = 0.01; S columns, *P* = 0.05; G2 columns, *P* = 0.03. C) Western blots showing the inactivation of SRC under overexpression of p19 either by the phosphorylation of Tyr527 or by decreasing the total SRC level (lanes pRK5-p19 compared to control lanes pRK5). A 3.5-fold less total SRC and 2.2-fold more p-SRC were quantified in lanes pRK5-p19 versus pRK5. As control the RACK1 level is also shown, which did not showed any notable change under p19 overexpression.

ERK1 and ERK2 are known to play different regulatory roles. For example, ectopic expression of ERK1 but not of ERK2 may act as a negative modulator of cell proliferation [19]. We showed here that ERK1 but not ERK2 is activated by p19 (Figure 1F). Additionally, activated ERK was recently reported to regulate G1/S delay [20]. To assess at which stage cell cycle progression could be modified by p19, we studied the cell phase distribution of HeLa cells overexpressing p19. Overexpression of p19 was observed to increase the number of cells in the G1 phase by 25% and prolong G1 phase length (Figure 2A and B). Interestingly, apoptosis was not observed in HeLa cells overexpressing p19, where no apoptotic peaks were detected; a finding that was further confirmed by the annexin/propidium iodure method and other protein markers, e.g. anti-PARP and eIF2α (our unpublished results). Thus, we conclude that p19 plays a role in regulating the switch from the G1 to S phase, thereby inducing a G1/S phase delay and also a decrease in the G2 phase. Therefore, the suggestion that ERK1 may act as a negative modulator of cell proliferation [19] in combination with our finding that activated ERK1 is also present during overexpression of p19 (Figure 1F), fits in well with the cell cycle progression data found for overexpression of p19 reported here.

The G1/S delay observed by p19 overexpression focused our attention on the SRC protein for three major reasons: (1) SRC is also a RACK-binding protein, (2) inactivation of SRC causes G1/S delay, and (3) RACK1 overexpression induces a partial arrest during the G1 phase by suppressing SRC activity at the G1 checkpoint [21]. Figure 2C shows that overexpression of p19 reduces SRC protein levels by 3.5-fold and inactivates SRC by increasing p-SRC(Tyr527) levels by 2.2-fold. Additionally, no change on RACK1 was observed (Figure 2C). This finding fits in well with our observation that overexpression of p19 induces G1/S phase delay.

### P19 Activates Telomerase through p73 Protein Binding

**Figure 3:**
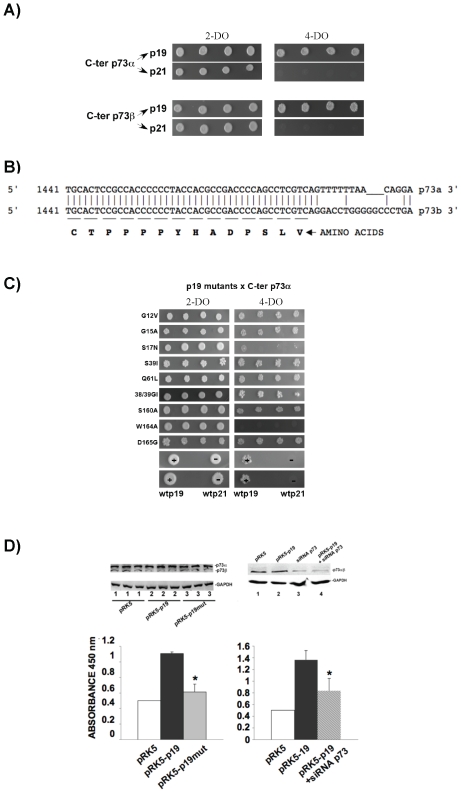
P19 interacts with the C-terminal of p73α/β and activates telomerase. A) Yeast two-hybrid assay on 4-DO plates to assess the binding potential of p19 and p21 to the C-terminal of p73α/β (C-ter p73α/β). P19 and p21 were cloned in the pGBKT7 vector and C-ter p73α/β in the pGADT7 vector. The vectors pGADT7-p73α and pGADT7-p73β contained 1441-1912 and 1441–1500 C-terminal nucleotides, respectively. Growth on 4-DO media indicates interaction between both proteins. Upper panels, interaction of p73α with p19 and p21; lower panels, interaction of p73β with p19 and p21. B) Nucleotide sequence comparison between p73α and p73β. C) Yeast two-hybrid assay of the binding potential of p19 mutants (G12V, G15A, S17N, S39I, Q61L, 38/39GI, S160A, W164A, D165G) with C-ter p73α grown on 2-DO and 4-DO yeast-selective plates (see Figure 1 legend) co-expressing pGBKT7-p19 mutants and pGADT7-p73α. Lower four panels are positive and negative controls (spotted twice), respectively: (+) wild-type p19×p73α on 2-DO (left) and 4-DO (right) and (−) wild-type p21×p73α on 2-DO (left) and 4-DO (right). D) Left graphic, telomerase activity assay in HeLa cells overexpressing p19: 1, empty vector; 2, p19; 3, p19mut. Telomerase activity is given under arbitrary units. The value of HeLa cells overexpressing empty vector was set to 0.5 and considered as basal telomerase activity (*P<0.001). Right graphic, HeLa cells were first transfected with the siRNA for p73 α/β (Santa Cruz Biotechnology) and incubated for 24 h in cell culture conditions after which the cells were then re-transfected with pRK5 or pRK5-p19 vector. Then, cells were harvested and telomerase activity was analyzed. The Figure shows that the RNAi of p73 α/β reverts the activation of telomerase by p19 (compare pRK5-p19 versus pRK5-p19+siRNA p73; *P<0.001). Above, control western blots showing that the overexpression of p19 does not alter p73 α/β levels (on the left) and that the p73α/β siRNA reduces p73 by 50% in lanes 3 and 4 as compared to lanes 1 and 2 on the right.

Previously, a yeast two-hybrid screen [11] has revealed putative p19-binding protein sequences, e.g. the C-terminal of p73α. More recently, a second isoform, p73β, has been described as a p19-binding protein [14]. All these protein-protein binding were previously confirmed by immunoprecipitation assays [11], [14]. Therefore, we tested binding of p73α and p73β to p19 and p21 using a yeast two-hybrid approach. We used the C-terminal of both p73 proteins (called C-ter p73α, from 1441–1912 nucleotides, and C-ter p73β, from 1441-stop codon nucleotides) that is known to contain the protein-binding site region. Figure 3A shows that p19 binds to both p73α and β, whereas p21 does not. This finding indicates that p73 interaction is p19 specific and that the p73 binding site should be in the common p73α/β region, i.e. amino acids from nucleotides 1441–1484 (see Figure 3B: CTPPPPYHADPSLV). Further testing of p19 with p53 (see Figure S2) revealed no binding. This finding is to be expected as p53 does not contain the CTPPPPYHADPSLV sequence found in p73. Testing a range of p19 mutants, C-ter p73α interaction was seen to be disrupted in W164A (see Figure 3C). Mutant S17N (Figure 3C) also disrupts binding to p73α, and this effect will be further studied in the future. We next investigated the co-localization of full-length p73α, and p19 was assayed *in vivo* in HeLa cells assays and observed co-localization inside the nucleus and perinuclear region (see Figure S3). Since RACK1 can also bind to p73, the results of the co-localization studies add further weight to the notion that RACK1, p19, and p73 may form a multimeric complex inside the nucleus, being this possible thus RACK1 and p73α have specific protein-protein binding sites distinct to the RACK1-p19 or p73α-p19 binding sites.

P73α and p73β have distinct functions [22], e.g. p73α is a strong inhibitor of telomerase, whereas p73β helps maintain telomerase activity through the activation of HDM2 and perhaps other targets [23]. As p19 binds to both p73α/β and telomerase is intrinsically linked to cell cycle progression, telomerase activity was assayed by overexpression p19 or p19mut in HeLa cells. Figure 3D shows that p19 increased telomerase enzymatic activity by 2 to 2.6-fold (columns pRK5-p19), but did not alter p73α/β protein levels (western blot in Figure 3D). In contrast, p19mut reverts the activation effect (column pRK5-p19mut). Taken together, we hypothesize that p19, probably through binding to both p73 isoforms, is an enhancer of the enzymatic telomerase activity. To test this hypothesis, p73 RNAi was obtained in the presence of overexpressed p19. This knock-down reduced the p73 level by 50% and additionally reverted the p19-induced telomerase activity (compare columns pRK5-p19 and pRK5-p19+siRNA p73 in the western blots of Figure 3D). These data indicate that p19 regulates telomerase activity through p73 protein binding.

### Cell Growth and Metastasic Genes Are Regulated by p19

To gain further insights into p19 function, the regulation of 22,000 different human genes in cells overexpressing p19 were quantified on microarrays, and results were validated in similar assays performed with cDNAs from HeLa cells transiently overexpressing p19mut. In both assays, basal expression of cDNAs from cells transiently transfected with empty vector was statistical set as fold change of 1 (no change). Table S1 lists all the genes that showed at least a 1.5-fold change between cells overexpressing p19 versus p19mut and Table S2 provides details on reported protein functions (array data in GEO with accession numbers GSE12059). The most striking observation of this assay is that very few genes vary their expression under conditions of overexpression of p19.

SLIT3 is known to regulate cell motility through Rac/Cdc42 activation [24]. In our assays with overexpression of p19, we observed a 2.8-fold increase in SLIT3 expression (see Table S1). This upregulation was re-validated by real-time PCR with SYBR-green dye and SLIT3 specific primers and was found to have a 5-fold increase with the overexpression of p19, that decreased to 3.9-fold with the overexpression of p19mut. C-Src expression decreased by 1.7-fold with overexpressed p19 (see Table S1) a result that was validated by western blot analysis (see Figure 2C).

Other genes were obtained with overexpression of p19mut and validated by statistical comparison with genes overexpressed with p19. Two main groups of proteins were found for overexpression of p19mut (Table S2): (1) chromatin structural proteins and (2) proteins that belong to and/or regulate the AP-1 transcription complex (c-Jun, ATF3, TRIB3, and TXNIP). ATF3 downregulation was confirmed by SYBR-green real-time PCR, showing no change with overexpression of p19 and a 2.2-fold decrease with p19mut. It is notable that many histone genes downregulated by p19mut belong to the same cluster (6p21.3 gene locus), indicating coordinated regulation at the transcriptional level.

Because c-Jun activation is regulated by RACK1 through direct RACK1-JNK binding [17], we paid special attention to the (−2.8-fold) downregulation of c-Jun induced by p19mut (see Tables S1 and S2). Western blot analysis of c-Jun protein levels in cells overexpressing p19 or p19mut revealed that higher levels of p19 increase c-Jun levels and p19mut decrease c-Jun levels even below basal levels (see Figure S4). This decrease of c-Jun level by p19mut is in line with the downregulation (−2.8-fold) observed in cells overexpressing p19mut shown with the microarray data (Table S1). No increase of p-c-Jun or p-JNK was found in HeLa cells overexpressing p19 (Figure 1F and results not shown), indicating that the observed higher c-Jun protein level does not equal c-Jun activation. Overexpression of human c-Jun without a concomitant increase of its phosphorylation was described in tumor cells of patients with classical Hodgkin's disease [25] and AML leukemia [26]. We therefore conclude that p19 regulates c-Jun at the transcriptional and protein level, but does not regulate p-Jun.

### Proteomic Profiling of p19 Overexpression Indicates a Regulatory Role in the TSC/mTOR Pathway

**Figure 4:**
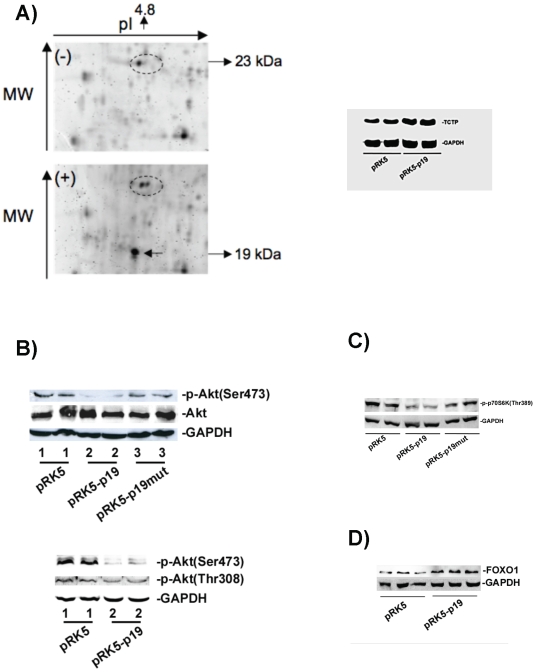
Proteins from the TSC/mTOR pathway are regulated by p19. A) 2D stained gel highlighting a representative region of the gel spots. (−) Negative control, proteins from HeLa cells transfected with empty vector; (+) proteins from HeLa cells overexpressing p19. The oval circle marks TCTP (pI 4.9 and 23 kDa) and the arrow shows overexpressed p19 (pI 4.8 and 19 kDa), which was also confirmed by microsequencing. Above, western blot analysis with anti-TCTP on proteins from HeLa cells transfected with empty vector or pRK5-p19, and a 1.6-fold increase was found for TCTP as quantified in lanes pRK5-p19 versus pRK5. Lane 1, proteins from cell transfected with empty vector; lane 2, proteins from cells transfected with p19. B) western blot analysis with anti-p-Akt (S473) and anti-p-AKT (Thr308) and anti-total Akt on proteins from HeLa cells transfected with empty vector (lanes 1), p19 (lanes 2), and p19mut (lanes 3). A decrease of both p-Akt (S473) and p-AKT (Thr308) by 50% were quantified in lanes pRK5-p19 versus pRK5. C) western blot analysis with anti-p-70S6K (Thr389) antibodies on proteins from HeLa cells transfected with pRK5, pRK5-p19, and pRK5-p19mut. A decrease of p-70S6K by 50% were quantified in lanes pRK5-p19 versus pRK5. D) western blot analysis with anti-FOXO1 antibodies of extracts from HeLa cells transfected with pRK5, pRK5-p19 and pRK5-p19mut. A 2-fold increase of FOXO1 was quantified in lanes pRK5-p19 versus pRK5.

HeLa cell proteins that changed their expression under transient overexpression of p19 were subjected to 2D gel analysis and proteins from spots representing up- or downregulated factors were microsequenced. Four spots were found to be upregulated by p19 overexpressed and microsequencing revealed them to be (1) TCTP (translationally controlled tumor protein, see the spot in Figure 4A; (2) the kinase nm23-H1 (metastasis inhibition factor nm23); (3) Profilin 1 (inhibitor of the polymerization of actin), and (4) PSMB6 (proteasome subunit). P19 (marked with an arrow in Figure 4A) was also identified and its digested peptides confirmed to be consistent with H-Ras p19. However, western blot analysis showed only TCTP to be upregulated and this was by 1.6-fold (Figure 4A). Recently, TCTP was suggested to have a GEF-like activity for Rheb [16], a RAS-related GTP-binding protein active in the TSC signaling pathway. Akt is a crucial player in this pathway, thus higher Akt activity inhibits TSC2/TSC1 GAP activity for Rheb, allowing mTOR to be activated [15]. We also found that p19 overexpression inhibited Akt phosphorylation on both Ser473 and Thr308 (see Figure 4B). Quantitative imaging revealed a 50% reduction of phosphorylated proteins. According to the observed TCTP upregulation and Akt hypophosphorylation, the p-70S6K(Thr389) from the mTOR pathway is hypophosphorylated (inactivated), with a 50% reduction of p-70S6K when p19 is overexpressed (see Figure 4C) as compared also with p19mut. Additionally, as hypophosphosrylation of Akt modulates the regulation of FOXO transcription factors [27], [28], we also observed that FOXO1 is upregulated in presence of p19 (Figure 4D). Collectively, these observations allowed us to conclude that p19 plays an inhibitory role in the mTOR pathway.

As many metastasis-related genes were observed to be up- or downregulated by p19 (see Tables S1 and S2) and 2D proteomic profiling highlighted that nm23-H1, a very important metastasis marker [29], could be a putative candidate for p19 regulation, the effect of p19 RNAi on the kinase nm23-H1 was studied. P19 RNAi was found to downregulate nm23-H1 protein expression (see Figure S5) This finding strongly points to a role for p19 as a putative metastasic protection factor.

## Discussion

### P19 Function and Pathways

#### PKC activation

Our data show that p19 activates PKC either by acting on RACK1-bound PKC via communication of an intra-complex or on soluble PKC directly. This PKC activation requires intact W164 amino acid on p19 H-Ras and this amino acid site is essential for 19 H-Ras to bind RACK1 and p73. SRC pathway. SRC is known to be highly regulated by and to bind to the RACK1 [21]. Our results show that SRC mRNA is downregulated and inhibited when p19 is overexpressed, whereas RACK1 showed no changes (Figure 2C). This finding correlates with the delay in the switch between the G1 and S phases under conditions of reduced SRC [21], as well as when p19 is overexpressed (Figure 2A and B). Therefore, p19 overexpression (Figure 2A and B) was also seen to have a similar effect as RACK1 overexpression [21], which induces a partial arrest during the G1 phase by suppressing SRC activity at the G1 checkpoint. MEK1/2 pathway. The finding that ERK1 phosphorylation is regulated by p19 is of key interest (Figure 1F). As p19 is known not to bind to Raf1 [11] and therefore making it unlikely that p19 can activate ERK via Raf1, then it is reasonable to suggest that ERK1 may be activated as a consequence of increased levels of PKC that leads to phosphorylation of Raf1 as well as other signals acting on ERK1/2 in the MEK1/2 pathway [30]. Our findings indicate that p19 can indirectly activate phosphorylation of ERK (Figure 1F) by first stimulating phosphorylation of PKC (Figure 1E). The ability of p19 to activate phosphorylated ERK is shared function with p21 [30]. P73/telomerase pathway. Both p73α and p73β bind to p19 -p73α in Figure 3A, reported in this work; p73β reported in [14]- while p53 does not (Figure S2). P73α is known to bind to RACK1, whereas p73β and p53 do not, and both p73α and p73β were reported to regulate telomerase activity [23]. Our findings that (1) p19 binds to both p73α and p73β, (Figure 3A), (2) overexpression of p19mut disrupts p73α/p19 interaction (Figure 3C), (3) p19, but not p19mut, increases telomerase activity (Figure 3D) and (4) knockdown of p73 in cells containing overexpressed p19 significantly reduced p19-induced telomerase activity, indicate suggest that p19 modulates the levels of available active p73α, and p73β. We therefore propose that p19 regulates telomerase activity by inhibiting p73α or activating p73β function towards hTERT. The c-Jun transcription factor. The up- and downregulation of c-Jun levels by p19 and its mutant, respectively (Tables S1 and S2), indicate that p19 regulates the AP-1 transcription complex. We found that higher activity of PKC driven by p19 (Figure 1E) does not lead to phosphorylation of JNK (another RACK1-binding protein), and consequently, the transcription factor c-Jun is also not phosphorylated (our unpublished results). These findings are in agreement with previous results [17] where the authors indicated that activation of PKC and its binding to RACK1 is not enough to activate JNK, and that a second stimulus is required in the MKK4/7 pathway [17]. Additionally, upregulation of c-Jun by p19 requires an intact W164 amino acid (Table S1). TSC pathway. Hypophosphorylation of Akt (S473) and (Thr308) indicates inhibition of the PI3K/insulin signaling pathway due to a lack of p-Akt [15]. The finding that ERK1 and Akt are hyper- and hypophosphorylated, respectively, in combination with an upregulation of TCTP, by overexpression of p19, is of key interest (Figure 1F, 4A and 4B. respectively) since all three proteins belong to the TSC pathway. The inhibition of the TSC pathway was verified by the inactivation of p70S6K by p19 overexpression; an effect that is reverted by the p19mut (Figure 4C).

### P19 Induces and Maintains a Reversible Quiescence State

**Figure 5:**
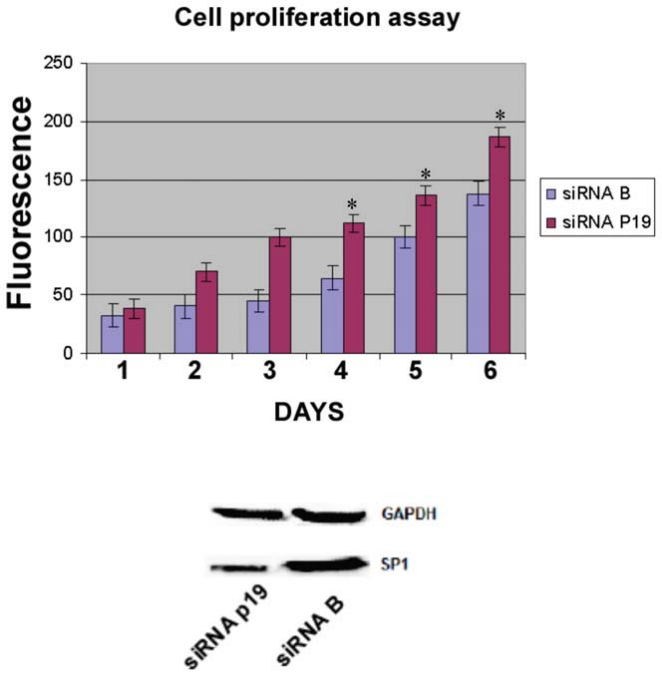
P19 RNAi induces cell proliferation. P19 RNAi was performed as detailed in methods. After three days of transfecting the p19 siRNA, cells were harvested and plated in 96 wells; 10.000 cells/well in 6 microplates by sixtuplicate and incubated at 37°C, 5% CO_2_ with DMEM/10% FCS. At the desired time, microplates were washed and freezed. Cells were quantified with the green fluorescent dye, CyQuant (Invitrogen) according provider instructions. Fluorescence measurements were made using a microplate reader with excitation at 485 nm and detection at 530 nnm. *P<0.003, as compared siRNA B with siRNA p19 on the same day. As negative control, the well tested negative siRNA B from Santa Cruz Biotechnology was also assayed in parallel in the same day microplate. Western blot below shows the siRNA of p19 compared with the negative control siRNA B and incubated with anti-GAPDH or anti-p19 antibody.

P19 overexpression induces G1/S phase delay; an observation that correlates with hypophosphorylation of Akt [31], [32]. Akt hypophosphorylation (at S473) has been observed in brain and muscle during squirrel hibernation, and Akt-driven mechanisms have been suggested to control the balance between cell proliferation and arrest [33]. A similar mechanism has been described in *C. elegans* where hypophosphosrylation of Akt modulates the regulation of FOXO transcription factors [27], [28], causing G1/S arrest that leads to a reversible cellular quiescence state [31], [32]. Similarly, we also observed that FOXO1 is upregulated when p19 is overexpressed (see Figure 4D). In fact, the FOXO1 level was doubled as can be seen when comparing the pRK5-p19 and the pRK5 lanes (Figure 4D). The three observations of (1) hypophosphorylation of Akt, (2) G1/S delay and (3) upregulation of FOXO1 lead us to conclude that p19 induces G1/S phase delay, thereby maintaining cells in a reversible quiescence state and preventing entry into apoptosis. Although 19 overexpression does not induce apoptosis, Kim *et al.* showed that p19 stimulates the p73β induced apoptosis when both proteins are simultaneously overexpressed [34]. The results here showed may explain this previous effect, thus there would be a combination of a cell arrest effect (induced by p19 overexpression) and cell arrest plus apoptosis effect (induced by p73β overexpression) that amplifies the synergistic activation of p73β-induced apoptosis [34]. In this direction we have assayed the effect of the p19 RNAi on the HeLa cell growth. As it can be seen in Figure 5, the reduction of p19 level increases the cell growth, having then just the opposite effect of a G1/S arrest or quiescence state.

Interestingly, TOR has been suggested to play a role in the quiescence mechanism in *S. cerevisiae* where inactivation of TORs leads to some characteristics of quiescence such as remodeled cell walls [35]. However, it should be noted that asimilar mTOR quiescence mechanism has not been described in mammals so far. This observation would also explain why few protein genes are up- and downregulated by p19 H-Ras overexpression. In summary: on one hand p19 induces FOXO1, that in combination with the G1/S phase delay, hypophosphorylation of both Akt and p70SK6 leads to maintenance of a reversible cellular quiescence state, thereby preventing entry into apoptosis. On the other hand, the p19 reduction induces cell growth.

## Methods

Human HeLa cells were used in all experiments and cultured in standard conditions (DMEM/10% FCS/5% CO_2_ and 37°C). Any growth specific treatment was applied to the cell culture. Transient overexpression assays were performed in triplicate in three independent experiments using the lipofectamine method (Invitrogen). Extracts and other assays were carried out after 24 h post-transfection unless otherwise stated in Figure legend. GAPDH protein was used as the internal loading control in all western blot assays.

### Yeast Two-Hybrid Assays

Pairwise protein-protein interactions were assayed in the yeast strain AH10(Clontech) after cotransformation with bait-and-prey constructs. Primary cotransformantwere selected on double drop-out media (-Leu/-Trp). To test interactions, we separately grew five independent Leu+/Trp+ colonies arising from each cotransformation experiment to saturation in (-Leu/-Trp) liquid medium, brought together, and 5 ml of this mixed culture were dropped on solid quadruple drop out medium (-Leu/-Trp/-His/-Ade). Colony growth was scored after 5 days of incubation at 30°C.

### RNA Interference Analysis with shRNA Vectors

The knockdown of p19 was performed by shRNA to the specific IDX nucleotide region. ShRNA vectors were constructed by cloning the following oligonucleotides in the pSUPER neo+gfp vector following manufacturer's instructions (OligoEngine):

pos_646.HIND: GATCCCCTCTGGCTCTAGCTCCAGCTTTCAAGAGAAGCTGGAGCTAGAGC CAGATTTTTA

pos_646.HIND_As:

AGCTTAAAAATCTGGCTCTAGCTCCAGCTTCTCTTGAAAGCTGGAGCTAGAGCCAGAGGG

P19 siRNA was performed with a specific siRNA duplex directed to the IDX nucleotide sequence: 5′ UCU GGC UCU AGC UCC AGC UTT 3′. The siRNA duplex was prepared 10 µM and 8 and 16 µl were transfected, with the Lipofectamine method, to HeLa cells during 3 days. See Figure 5 legend for additional information.

After transfection, HeLa cells were harvested after 3 days to obtain protein and RNA extracts. A 60% decrease of p19 mRNA was observed by Taqman real-time PCR (with E3-IDX Taqman assay), while p21 did not show changes on the mRNA level (with E3-E4A Taqman assay). Therefore we conclude that the p19 RNAi is only directed to p19 and not to p21 mRNAs.

See Supplementary Methods S1 and Figures legends for additional information regarding transfections, other constructs, immunufluorescenece studies, ESTs microarray profiling, FACS studies, yeast two-hybrid assays, enzymatic assays, proteomic profiling, p73 siRNA, analysis of mRNA expression, gene expression analysis and bioluminescence imaging image quantification. This work does not require an ethics statement.

## Supporting Information

Supplementary Methods S1 (0.10 MB DOC)

Figure S1 Co-localization of p19 and PKCβII. P19 and PKCβII were transiently overexpressed in HeLa cells and detected by indirect IF with specific antibodies raised in rabbit and mouse, respectively. Secondary antibodies were Alexa Fluor® 488 F(ab')2 labeled anti-rabbit (p19 image) or Alexa Fluor® 555 F(ab')2 labeled anti-mouse (PKCβII image), respectively. Merge image shows the co-localization of the Alexa Fluor® 488 and Alexa Fluor® 555 secondary antibodies.
(0.18 MB TIF)

Figure S2 Yeast two-hybrid assay in DDO and QDO plates of the potential binding of p19 H-Ras to p53. P19 H-Ras was cloned in pGBKT7 vector and p53 (full-length) was in pGADT7 vector.
(0.60 MB TIF)

Figure S3 Co-localization of p19 H-Ras and p73α. P19 and p73α (complete sequence) were transiently overexpressed in HeLa cells and detected by indirect IF with specific antibodies raised in rabbit and mouse, respectively. Secondary antibodies were Alexa Fluor® 488 F(ab')2 labeled anti-rabbit (p19 image) or Alexa Fluor® 555 F(ab')2 labeled anti-mouse (p73α image), respectively. Merge image shows the co-localization of the Alexa Fluor® 488 and Alexa Fluor® 555 secondary antibodies. Upper and lower panels show two different co-localizations of the samples.
(2.31 MB TIF)

Figure S4 Western blot analysis of total JUN protein levels in two independent experiments HeLa cells overexpressing empty vector (lane 1), p19 (lane 2), and p19mut (lane 3). Anti-JUN, anti-GAPDH (internal control), and anti-p19 (detecting both wild-type and mutant p19) were the selected antibodies.
(0.51 MB TIF)

Figure S5 A) 2-D stained gel highlighting two sequence spots, as example of three independent experiments. (−) Negative control, proteins from HeLa cells transfected with empty vector; (+) proteins from HeLa cells overexpressing p19. Oval circle marks nm23-H1 (pI 5.8 and 17 kDa) and arrow shows overexpressed p19 (pI 4.8 and 19 kDa), which was also confirmed by microsequencing. Western blots from the same samples did not show a clear overexpression of nm23-H1; however RNAi of p19 (showed in B) showed a clear decrease on the nm23-H1 protein level, indicating that p19 regulates nm23-H1.
(0.11 MB TIF)

Table S1 Two upper tables: Log2 is the log2 value of the fold change measuring overexpression of pRK5-p19 as compared first to pRK5 empty vector and then compared to overexpression of pRK5-p19mut in the collection of ESTs microarrays. Two lower tables: Log2 is the log2 value of the fold change measuring overexpression of pRK5-p19mut as compared first to pRK5 empty vector and then compared to overexpression of pRK5-p19. RT indicates confirmation by Real-time PCR and WB confirmation by Western blot (** P<0.001).
(0.13 MB DOC)

Table S2 * Putative effect of the p19 H-Ras on cancer progression. ↑ increasing of mRNA expression ↓ decreasing of mRNA expression
(0.08 MB DOC)
