# Supplementary material for: P19 H-Ras Induces G1/S Phase Delay Maintaining Cells in a Reversible Quiescence State
Source: PLoS One. 2009 Dec 30;4(12):e8513. doi: 10.1371/journal.pone.0008513 (PMC2798614; doi:10.1371/journal.pone.0008513)
Supplement: Supplementary Methods S1 — (0.10 MB DOC) [file pone.0008513.s001.doc]

**Supplemental Experimental Procedures**

**Cell Culture, Transfections, Antibodies, and Western Blots**

Other cell lines for the indirect immunofluorescence (IF) was cultured according to ATCC protocols. HeLa cell transfections were performed with lipofectamine/plus reagent according Invitrogen’s standard protocols. SP1 anti-p19 was produced as previously described (Guil et al., 2003a). SP57 anti-p19 was directed in a rabbit towards the p19 C-terminal and only recognizes only high levels of denatured p19 on SDS gels (1/500) and recognizes native p19 by indirect IF (1/1000). All other antibodies were purchased: mouse anti-p73 (Ab-4 cocktail, Lab Vision Corporation); mouse anti-PKCβII (Santa Cruz Biotech), anti-JUN (Cell Signaling); anti-GAPDH (Abcam); anti-TCTP (Eurogentec) anti-nm23 (Santa Cruz Biotech.); anti-p-eIF2α S52 (Biosource); anti-cleaved Notch-1 (Cell Signaling); anti-p-ERK1/2 T202/Y204 and anti-ERK1/2 (Cell Signaling); anti-Akt (Cell Signaling); anti-p-Akt T308 and anti-p-Akt S473 (Santa Cruz Biotech.); anti-p-p70 S6 kinase T389 (Cell Signaling); anti-p-Jun S63 and anti-p-Jun S73 (Cell Signaling); anti-p-p38 MAPK T180/Y182 (Cell Signaling); anti-p-SAPK/JNK T183/Y185 (Cell Signaling); anti-SRC and anti-p-SRC Tyr527 (Cell Signaling) and anti-FOXO1. Antibodies dilutions were performed according to each manufacturer’s instructions. Protein extraction for SDS gels and Western blots was essentially performed as previously described (Guil et al., 2003a). Chemiluminescence on western blots was quantified by the imaging software Multi Gauge V3.0.

**IF and IF Co-Localizations.**

HeLa cells were seeded onto coverslips the day before transfection. Transfection was performed as stated above. After overnight incubation, cells were fixed with 4% paraformaldehyde and washed twice in PBS, and the coverslips were mounted on glass slides as described previously. After paraformaldehyde fixation, cells were treated with NaBH4 (1 mg/ml), permeabilized with 0.2% (v/v) Triton X-100, and blocked with 3% BSA-PBS. p19 expression was visualized by use of either the crude SP57 serum.

Double staining of the cells with SP57 and anti-p73 or anti-PKCβII were described in Figure legends. Primary antibodies were detected either by TRITC-labeled anti-rabbit or FITC-labeled anti-mouse antibodies. Fluorescence images were obtained with a Leica TCS 4D (Leica Lasertechnik) confocal scanning laser microscope adapted to an inverted Leitz DMIRBE microscope and a X63/1.4 oil Plan-Apo objective. The light source was an argon/krypton laser (75 mW). Green fluorescence from FITC-conjugated secondary antibodies and red fluorescence from TRITC-conjugated secondary antibodies were excited at 488 and 568 nm, respectively. Optical sections of 0.5 μm were obtained, and colocalization analysis was performed with Metamorph Imaging System software (version 3.5; Universal Imaging Corporation).

**Enzymatic Activities**

Telomerase activity was determined by using the TeloTAGGG Telomerase PCR ELISA kit (Roche Applied Science) according to the manufacturer’s instructions (<http://www.roche-applied-science.com/pack-insert/2013789a.pdf>). In brief, Elongation/

Amplification: In the first step, telomerase adds telomeric repeats (TTAGGG) to the 3’-end of the biotin-labeled synthetic P1-TS-primer. These elongation products, as well as the Internal standard (IS) included in the same reaction vessel, are amplified by PCR using the primers P1-TS and the anchor-primer P2. PCR products

derived from telomerase-mediated elongation products in the first step contain the telomerase-specific 6 nucleotide increments, while the Internal standard (IS) generates a 216 bp PCR product. Detection by ELISA: The PCR products are split into two aliquots, denatured and hybridized sepa-rately to digoxigenin-(DIG)-labeled detection probes, specific for the telomeric repeats (P3-T) and for the Internal standard (IS) (P3-Std), respectively. The resulting products are immobilized via the biotin label to a streptavidin-coated microplate. Immobilized amplicons are then detected with an antibody against digoxigenin that is conjugated to horseradish peroxidase (Anti-DIG-HRP) and the sensitive peroxidase substrate TMB and read at 450 nm. PKC-specific activity was determined using the MESACUP Protein Kinase Assay Kit (MBL, Medical & Biological Laboratories CO., LTD) according to the manufacturer’s instructions ([www.mblintl.com/mbli/catalog/pdf/5230.pdf](http://www.mblintl.com/mbli/catalog/pdf/5230.pdf)). In brief, the non-radioactive method for measuring PKA/PKC is based on ELISA that utilizes a synthetic peptide (RFARKGSLRQKNV), bound to the microplate, and a biotinylated monoclonal antibody recognizing phosphorylated form of the peptide. Biotin is then recognized by peroxidase conjugated streptavidin, and after adding substrate, the ELISA is finally read at 492 nm.

**Microarrays**

CDNA samples from HeLa cells transfected with pRK5 vector encoding either the wild-type form of p19 or p19mut were compared against control samples transfected with empty vector pRK5. Two-color hybridizations were performed in duplicate with dye reversal. A total of 12 human 1A v2 oligonucleotide microarrays from Agilent (G4110B) bearing over 22,000 probes were hybridized according to the manufacturer's recommended procedures: 6 wt p19 versus control and 6 p19W164A versus control. Briefly, 500 ng of total RNA from each sample were amplified by Oligo-dT-T7 reverse transcription and labeled by *in vitro* transcription with T7 RNA polymerase in the presence of Cy5-CTP or Cy3 CTP using the Low Input RNA labeling kit (Agilent) and cleaned using RNAeasy columns (Qiagen). After fragmentation, 750 ng of labeled cRNA from each of two samples were co-hybridized in in situ hybridization buffer (Agilent) for 18 h at 60ºC and washed for 10 min in 6X SSC + 0.005% Triton X-102 at room temperature, and 5 min in 0.1X SSC + 0.005% Triton X-102 at 4ºC, followed by drying by centrifugation for 5 min at 1000 rpm. Images were generated on an Agilent confocal microarray scanner at 10 µm resolution and quantified using GenePix 6.0. Spots with signal intensities twice above the local background that were also not saturated or flagged by GenePix were considered reliable and with a weight of 1 for normalization purposes, whereas the rest were given weights of 0.01. Extracted intensities were subtracted from the local background and the log_2_ ratios were normalized in an intensity-dependent fashion by the global lowess method with a span parameter of 0.3. Normalized log_2_ ratios were scaled between arrays to allow better comparison of data. Raw data were processed using MMARGE (Lozano et al, unpublished), a web implementation of Limma (Smyth et al., 2005), and a microarray analysis library developed within the Bioconductor project in the R statistical environment (Gentleman et al., 2004). For determining significant hits, the empirical Bayes statistic B was computed. To compare the wt and mutated forms, a contrast test was applied taking the normalized log_2_ ratios of the six microarray hybridizations of each type as replicates against the same common control reference using BANAL, a web-based interface running also on Limma (Lozano et al, unpublished).

**Determination of Cell Cycle Phase Percentages.**

HeLa cells were transfected with different plasmid DNA encoding a GFP sequence as described previously (Guil et al., 2003a). Cells were incubated with 2 μg/ml Hoechst 33342 for 2 h. Prior to analysis, cells were washed and resuspended at 1 x 10^6^ cells/ml in DMEM supplemented with 10% FBS, penicillin-streptomycin, and Hoechst. Flow cytometric experiments were carried out using a MoFlo flow cytometer (DakoCytomation, Fort Collins, Colorado, USA). Excitation of the samples was done using a Coherent Enterprise II argon-ion laser. Excitation with the blue line of the laser (488 nm) permits the acquisition of forward scatter (FS), side scatter (SS), and green (530 nm) fluorescence from GFP. UV emission (20 W) was used to excite Ho33342 blue fluorescence (450 nm). Doublets were discriminated using an integral/peak dot plot of Hoechst fluorescence. Optical alignment was based on optimized signal from 10 μm fluorescent beads (Flowcheck, Coulter Corporation, Miami, Florida, USA).

**RNA Extraction and RT-PCR**

Total cellular RNA was extracted from HeLa cells using the NucleoSpin® RNA II Kit (Machenerey-Nagel, BD Biosciences), and for the real-time PCR an additional DNAse step was performed using DNAse enzyme (RQ1 RNase-free DNase, Promega). cDNA was synthesized using SuperScript^TM^ III First-Strand Synthesis SuperMix for qRT-PCR Reverse Transcriptase (Invitrogen).

**Plasmid Constructions**

All plasmid inserts were obtained by PCR using *Pfu* DNA polymerase with specific oligodeoxynucleotide primers (see below) All constructs were verified by DNA sequencing.

**Proteomic Analysis**

Protein extracts were obtained from HeLa cells by using the Sample Grinding Kit (Amersham Biosciences) and 2-D Clean-up Kit (Amersham Biosciences). Before proteomic analysis, samples were cleaned-up using a 2D Clean-up kit (GE Healthcare, Amersham, England) according to the manufacturer’s guidelines. The resulting protein pellets were resuspended in 7M Urea, 2M Thiourea, 4% (w/v) CHAPS and 225 μg of each sample was made up to 450µl with rehydration buffer (7M urea, 2M thiourea, 4% [w/v] CHAPS, 0.5% [v/v] IPG Buffer pH3-11NL and 0.002% [w/v] bromophenol blue) and loaded onto a 24 cm Immobiline DryStrip gel (pH 3–11 non-linear) by passive rehydration for a minimum of 12h. Following rehydration, the DryStrip gel was transferred to an Ettan IPGPhor system (GE Healthcare, Amersham, England) and isoelectric focusing was performed by applying 500 V for 1h, 1,000 V for 1h, and 8,000 V for 10.5h until a total of 64,000 Vh had been achieved. After isoelectric focusing, strips were equilibrated in SDS equilibration buffer (50 mM Tris-HCl, pH 8.8, 6 M urea, 30% [v/v] glycerol, 2% [w/v] SDS, and 0.002% [w/v] bromophenol blue) containing 1% [w/v] DTT for 15 min at room temperature followed by an incubation in SDS equilibration buffer containing 2.5% [w/v] iodoacetamide for 15 min at room temperature. After equilibration, strips were applied to 12.5% (w/v) SDS-PAGE gels and proteins separated in the second dimension using the Ettan DALT ll separation unit (GE Healthcare, Amersham, England) at 0.2W/gel for 1h, 0.4W/gel for 1h and then 20W/gel until completion. Gels were stained using SYPRO ruby total protein gel stain, according to the manufacturer’s instructions (Molecular Probes, Invitrogen, Paisley, Scotland) and imaged using a Typhoon 9400 variable mode imager (GE Healthcare, Amersham, England).

**Spot Picking, Protein Processing and Mass Spectrometry**

Selected protein spots were cut from the gel, using the Investigator ProPic Automated 2D spot picker and digested with trypsin using the ProGest automated digestion unit (both from Perkin Elmer Life Sciences (UK) Ltd., Beaconsfield, England). The resulting peptides were analysed by mass spectrometry using a 4700 MALDI-TOF/TOF mass spectrometer (Applied Biosystems, Foster City, CA) to give a peptide mass fingerprint and peptide sequence information, which was searched against various databases using the Mascot search program (www.matrixscience.com) to identify the protein present in the gel spot.

**Apoptosis Assays**

HeLa cells were transfected with the pRK5-p19 plasmid as indicated in material and methods. Apoptotic cells were detected by flow cytometry using the Annexin V-FITC Apoptosis Detection Kit (Sigma) according to the manufacturer's instructions. Flow cytometric experiments were carried out using an Epics XL flow cytometer (Coulter Corporation, Miami, Florida, USA). Excitation of the samples was achieved by using a air-cooled argon-ion laser set at 488 nm and 15 mW power with other parameters at standard configuration. Forward scatter (FSC), side scatter (SSC), green (525 nm) fluorescence for Annexin-FITC-conjugated antibody, and red (675 nm) fluorescence for PI. Green fluorescence was collected with a 550 nm dichroic-long filter and 525 nm band pas filter. Red fluorescence was collected with a 645 nm dichroic-long filter and a 675 nm band pass filter. Optical alignment was based on optimized signal from 10 nm fluorescent beads (Immunocheck, Epics Division). Apoptotic (FITC), death cells (IP-labeled), and non-altered (i.e. neither annexin nor PI) populations were quantified simultaneously on FITC/IP dot plots.

**Taqman Assays and SYBR-Greeen Real-Time PCR**

TaqMan primers for exons E3-IDX (Hs00978053_g1), E4A-E4B (Hs00978051_g1,) and E3-E4A (Hs00610483_m1) and for GAPDH (Hs99999905_m1, housekeeping) were supplied by Applied Biosystems Gene Expression Assays (Applied Biosystems). Assays were run with TaqMan® Universal PCR Master Mix, No AmpErase® UNG (uracil-N-glycosylase) on a 7000 Sequence Detection System ABI PRISM (Applied Biosystems) using universal cycling conditions (10 min at 95ºC, 15 sec at 95ºC, 1 min at 60ºC, 40 cycles). C_T_ values from each reaction were determined of a fixed threshold value (0.4), and the final C_T_ values employed were the average of three replicates. Calculations were performed according to the ΔΔC_T_ method and respective GAPDH values (User Bulletin #2 ABI Prism 7700 Sequence Detection System, December 11, 1997). SYBR-green real-time PCR was performed in universal conditions, as described above, but with the following oligonucleotides: ATF3dir: 5' AGC CTG GAG CAA AAT GAT GC 3'; ATF3rev: 5' TCC GAG GCA GAG ACC TGG 3'; SLIT3dir: 5' TGC CTG GGA CAA GTA GTC CG 3'; SLIT3rev: 5' TGC ATA ACC TTT CTG GCG G 3'.

**Oligodeoxynucleotides for Plasmid Constructs**

**pEGFP-C1-IDX**

kpn2IIDXdir (5’-AAG TCC GGA GGC AGC CGC TCT GGC TCT-3’)

IDXBamHIrev (5’-GGT GGA TCC TCA CAT GGG TCC CGG GGG-3’)

**pEGFP-C1-p19 (E1-E3)**

kpn2Ip19dir (5’-AAG TCC GGA ATG ACG GAA TAT AAG CTG-3’)

E3(p19)BamHIrev (5’-GGT GGA TCC TCA CG CCG GGT CTT GGC-3’)

**pEGFP-C1-RACK1**

kpn2IRACK1dir (5’-AAG TCC GGA ATG ACC GAG CAA ATG ACC-3’)

RACK1Smarev (5’-GGG GCG GGT ACC AAT AGT CAC CTG C-3’)

**pEGFP-C1-RACK1-IDX**

We inserted IDX in the pEGFP-C1-IDX vector

**pRK5-p19 W164A**

EcoRIp19W164Adir (5'-TCT GAA TTC ATG ACG GAA TAT AAG CTG G-3')

p19W164ASalIrev (5'-GAG GTC GAC TCA CAT GGG TCC CGG GGG G-3')

**pRK5-p73α**

HindIIIp73αdir (5'-ATC AAG CTT ATG GCC CAG TCC ACC GCC A-3')

p73αSalIrev (5'-GAG GTC GAC TCA GTG GAT CTC GGC CTC C-3')

**pRK5-PKCβII**

XbaIPKCβIIdir (5'-TCC TCT AGA ACA TGG CTG AC-3')

PKCβIISalIrev (5'-ACC GTC GAC TTA GCT CTT GA-3')

**pSUPER gfp+neo RNAi 712**

RNAi 712 dir (5'-GAT CCC CTC GCG CTG GAG TGG AGG ATT TCA AGA GAA TCC TCC ACT CCA GCG CGA TTT TTA-3')

RNAi 712 rev (5'-AGC TTA AAA ATC GCG CTG GAG TGG AGG ATT CTC TTG AAA TCC TCC ACT CCA GCG CGA GGG-3')

**pSUPER gfp+neo RNAi 646**

RNAi 646 dir (5'-GAT CCC CTC TGG CTC TAG CTC CAG CTT TCA AGA GAA GCT GGA GCT AGA GCC AGA TTT TTA-3')

RNAi 646 rev (5'-AGC TTA AAA ATC TGG CTC TAG CTC CAG CTT CTC TTG AAA GCT GGA GCT AGA GCC AGA GGG-3')

**pEYFP-C1-p19**

kpn2Ip19dir (5’-AAG TCC GGA ATG ACG GA TAT AAG CTG-3’)

p19BamHIrev (5’-GGT GGA TCC TCA CAT GGG TCC CGG GGG-3’)

**pECFP-C1-RACK1**

kpn2IRACK1dir

RACK1bluntrev (5’-TCA GCG GGT ACC AAT AGT CAC CTG CCA-3’)

**pECFP-C1-p73α**

Kpn2Ip73αdir (5'-CTC TCC GGA ATG GCC CAG TCC ACC G-3')

p73αSalIrev (5'-ACC GTC GAC TCA GTG GAT CTC GGC C-3')

**pGADT7-p120GAP (714-1047 AA)**

SmaIp120GAP714-1047dir (5’-GAT GGA AAA AAT CAT GCC ACA ACA A-3’)

p120GAP714-1047rev (5’-CTA CCT GAC ATC ATT GGT TTT TGT A-3’)

**pGADT7-yCdc25 (2721-4770 nt)**

EcoRIyCdc25 2721-4770dir (5’-AGT GAA TTC ATG TCT TCG GTC TCC T-3’)

yCdc25 XhoIrev (5’-CTG CAG CTC GAG TTA TCG AAA TAA C-3’)

**pGADT7-SOS (564-1049 AA)**

SOS564-1049SalIdir (5’-CAG GTC GAC CGG TAC CTG GTC TTG G-3’)

SmaISOS564-1049rev (5’-CCA CCC GGG TGA GGA GCA GAT GAG G-3’)

**pGADT7-PI3K (110α)**

NdeIPI3K110adir (5'-GCT CAT ATG CCT CCA AGA CCA TCA TCA G-3')

PI3K110a XhoIrev (5'-TCA TCA CTC GAG TCA GTT CAA AGC ATG C-3')

**pGADT7-p73α (1441-1912nt)**

p73α (1441-1912)dir (5'-CTG CAC TCC GCC ACC CCC CTA CCA C-3')

p73α (1441-1912)rev (5'-TCA GTG GAT CTC GGC CTC CGT GAA C-3')

**pGADT7-p73β (1441-1500nt)**

p73α (1441-1912)dir (5'-CTG CAC TCC GCC ACC CCC CTA CCA C-3')

p73β (1441-1500)rev (5'-TCA GGG CCC CCA GGT CCT GAC GAG-3')

**REFERENCES**

Gentleman, R. C., Carey, V. J., Bates, D. M., Bolstad, B., Dettling, M., Dudoit, S., Ellis, B., Gautier, L., Ge, Y., Gentry, J.*, et al.* (2004). Bioconductor: open software development for computational biology and bioinformatics. Genome Biol *5*, R80.

Guil, S., de La Iglesia, N., Fernandez-Larrea, J., Cifuentes, D., Ferrer, J. C., Guinovart, J. J., and Bach-Elias, M. (2003a). Alternative splicing of the human proto-oncogene c-H-ras renders a new Ras family protein that trafficks to cytoplasm and nucleus. Cancer Res *63*, 5178-5187.

Guil, S., Gattoni, R., Carrascal, M., Abián, J., Stévenin, J., and Bach-Elias, M. (2003b). Roles of hnRNP A1, SR proteins, and p68 Helicase in

c-H-ras Alternative Splicing Regulation. Mol Cel Biol *23*, 2927–2941.

Smyth, G. K., Michaud, J., and Scott, H. S. (2005). Use of within-array replicate spots for assessing differential expression in microarray experiments. Bioinformatics *21*, 2067-2075.
