# Supplementary figures and images for: P19 H-Ras Induces G1/S Phase Delay Maintaining Cells in a Reversible Quiescence State
Source: PLoS One. 2009 Dec 30;4(12):e8513. doi: 10.1371/journal.pone.0008513 (PMC2798614; doi:10.1371/journal.pone.0008513)

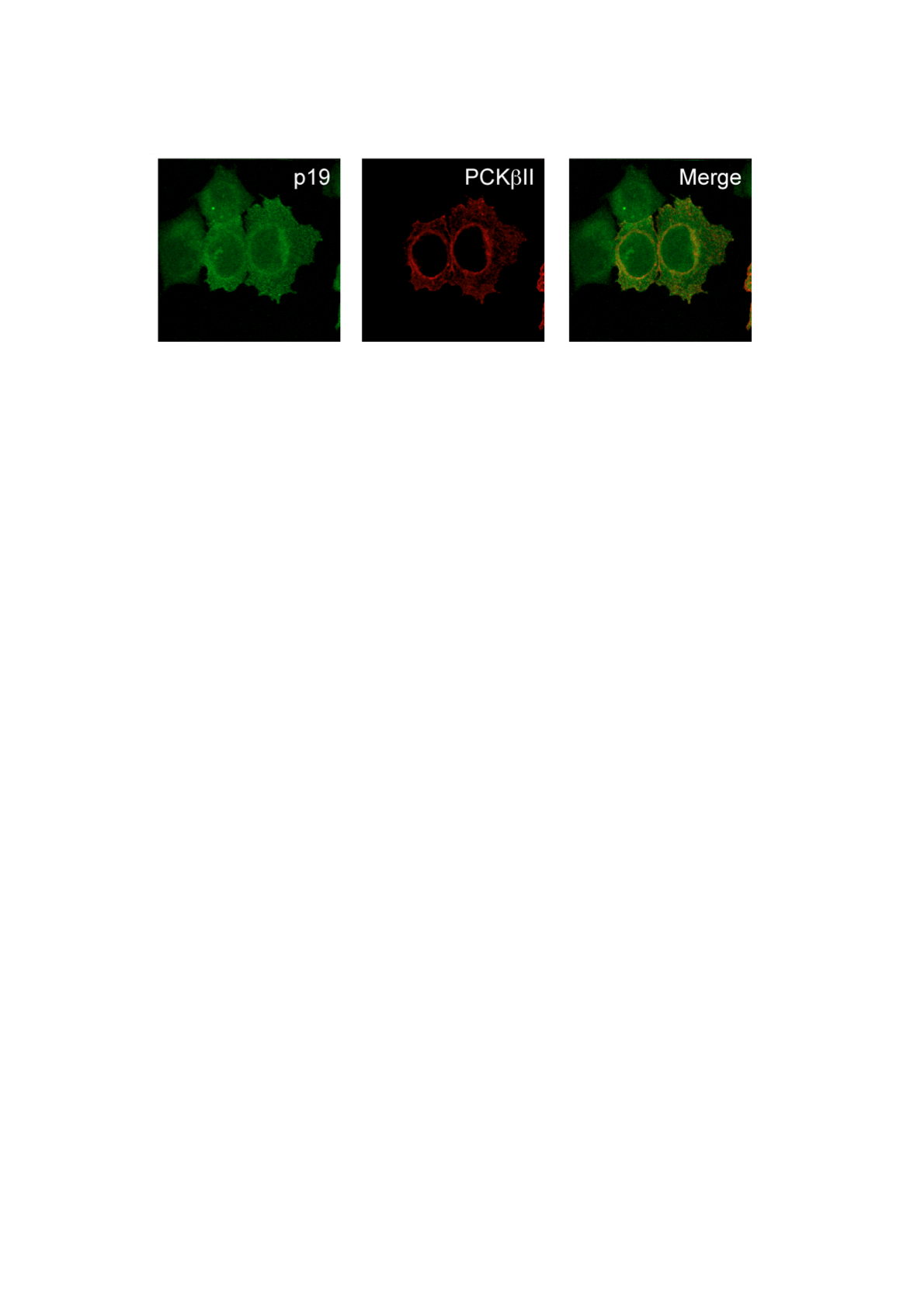

Supplement: Figure S1 — Co-localization of p19 and PKCβII. P19 and PKCβII were transiently overexpressed in HeLa cells and detected by indirect IF with specific antibodies raised in rabbit and mouse, respectively. Secondary antibodies were Alexa Fluor® 488 F(ab')2 labeled anti-rabbit (p19 image) or Alexa Fluor® 555 F(ab')2 labeled anti-mouse (PKCβII image), respectively. Merge image shows the co-localization of the Alexa Fluor® 488 and Alexa Fluor® 555 secondary antibodies. (0.18 MB TIF) [file pone.0008513.s002.tif]

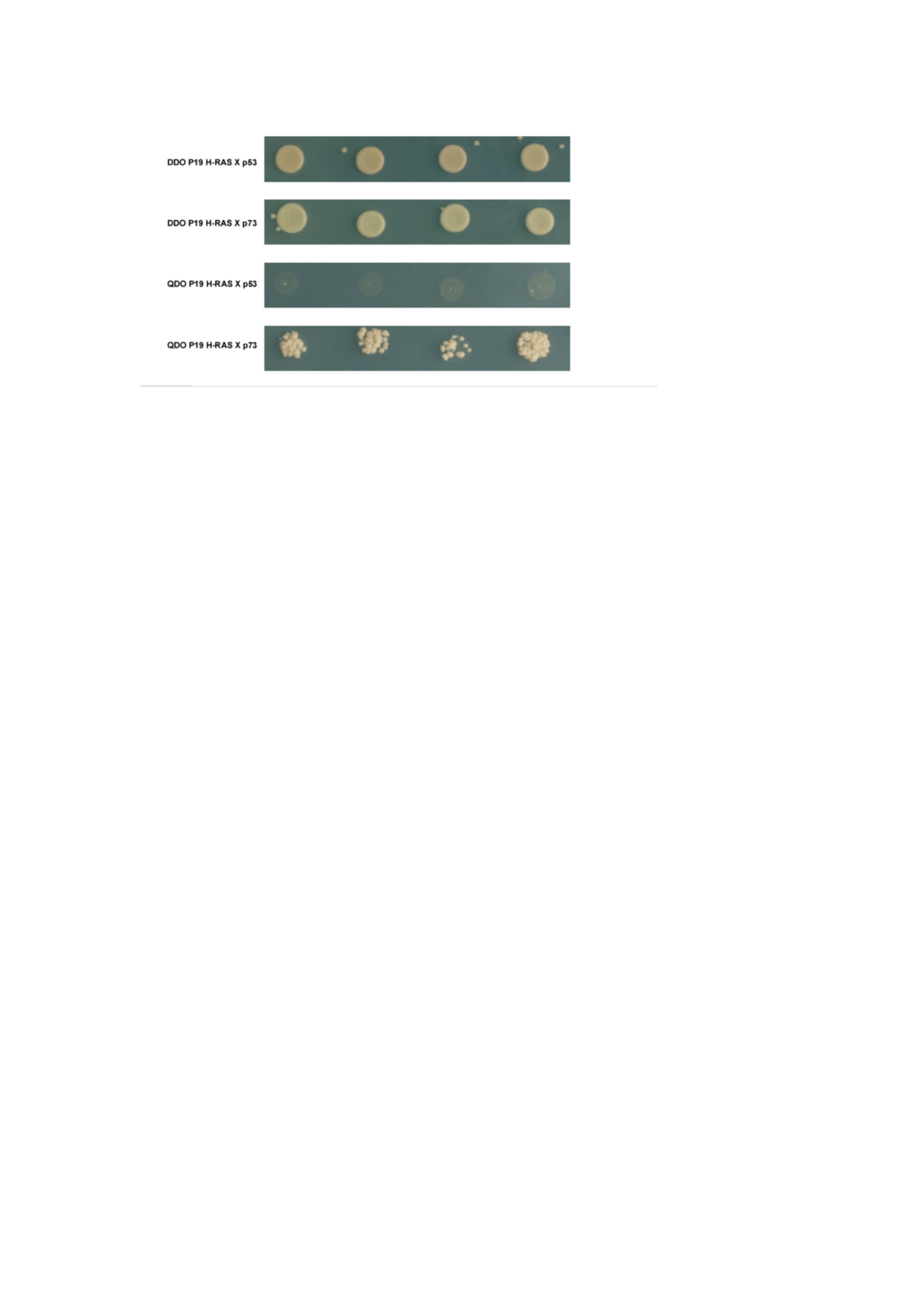

Supplement: Figure S2 — Yeast two-hybrid assay in DDO and QDO plates of the potential binding of p19 H-Ras to p53. P19 H-Ras was cloned in pGBKT7 vector and p53 (full-length) was in pGADT7 vector. (0.60 MB TIF) [file pone.0008513.s003.tif]

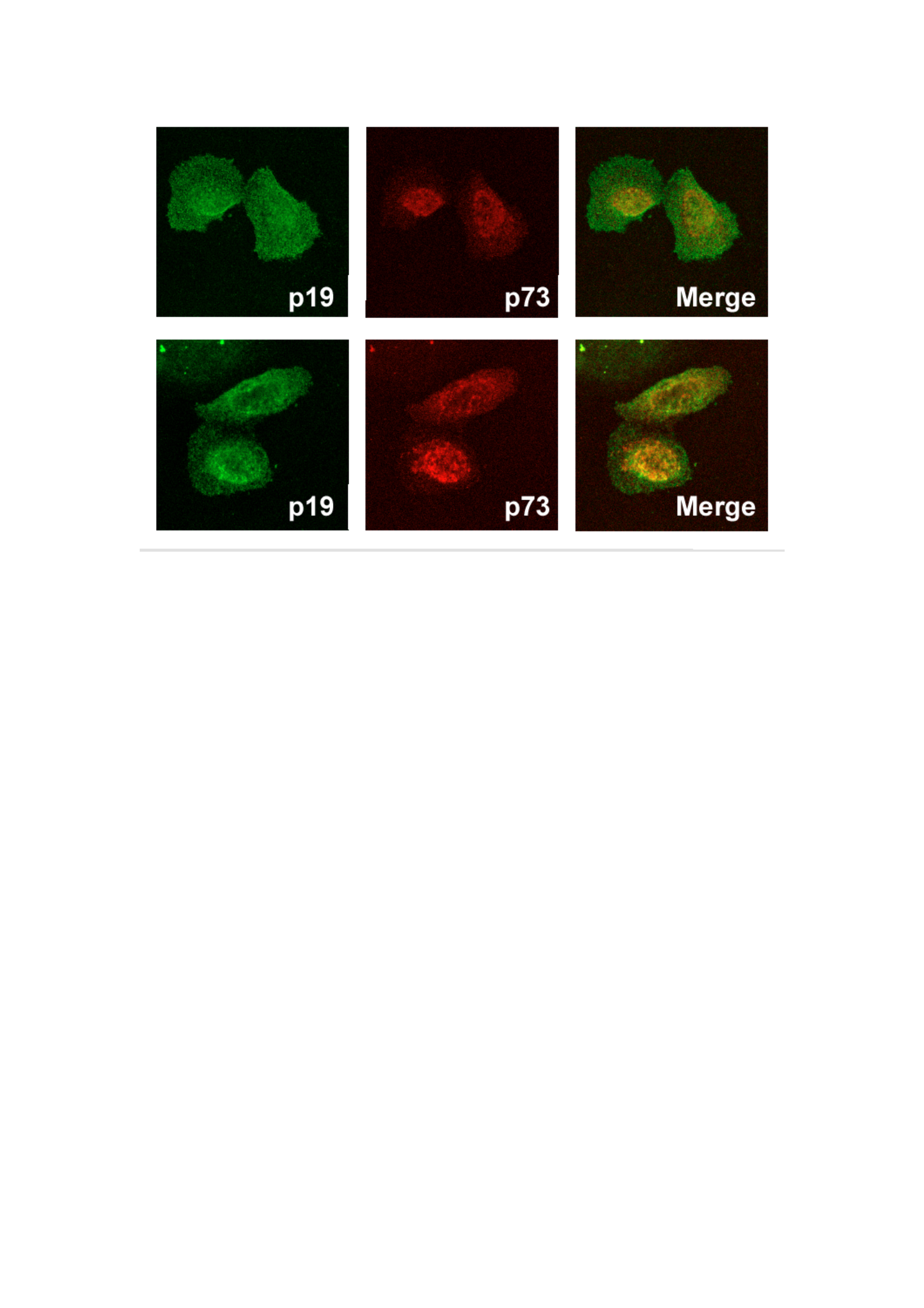

Supplement: Figure S3 — Co-localization of p19 H-Ras and p73α. P19 and p73α (complete sequence) were transiently overexpressed in HeLa cells and detected by indirect IF with specific antibodies raised in rabbit and mouse, respectively. Secondary antibodies were Alexa Fluor® 488 F(ab')2 labeled anti-rabbit (p19 image) or Alexa Fluor® 555 F(ab')2 labeled anti-mouse (p73α image), respectively. Merge image shows the co-localization of the Alexa Fluor® 488 and Alexa Fluor® 555 secondary antibodies. Upper and lower panels show two different co-localizations of the samples. (2.31 MB TIF) [file pone.0008513.s004.tif]

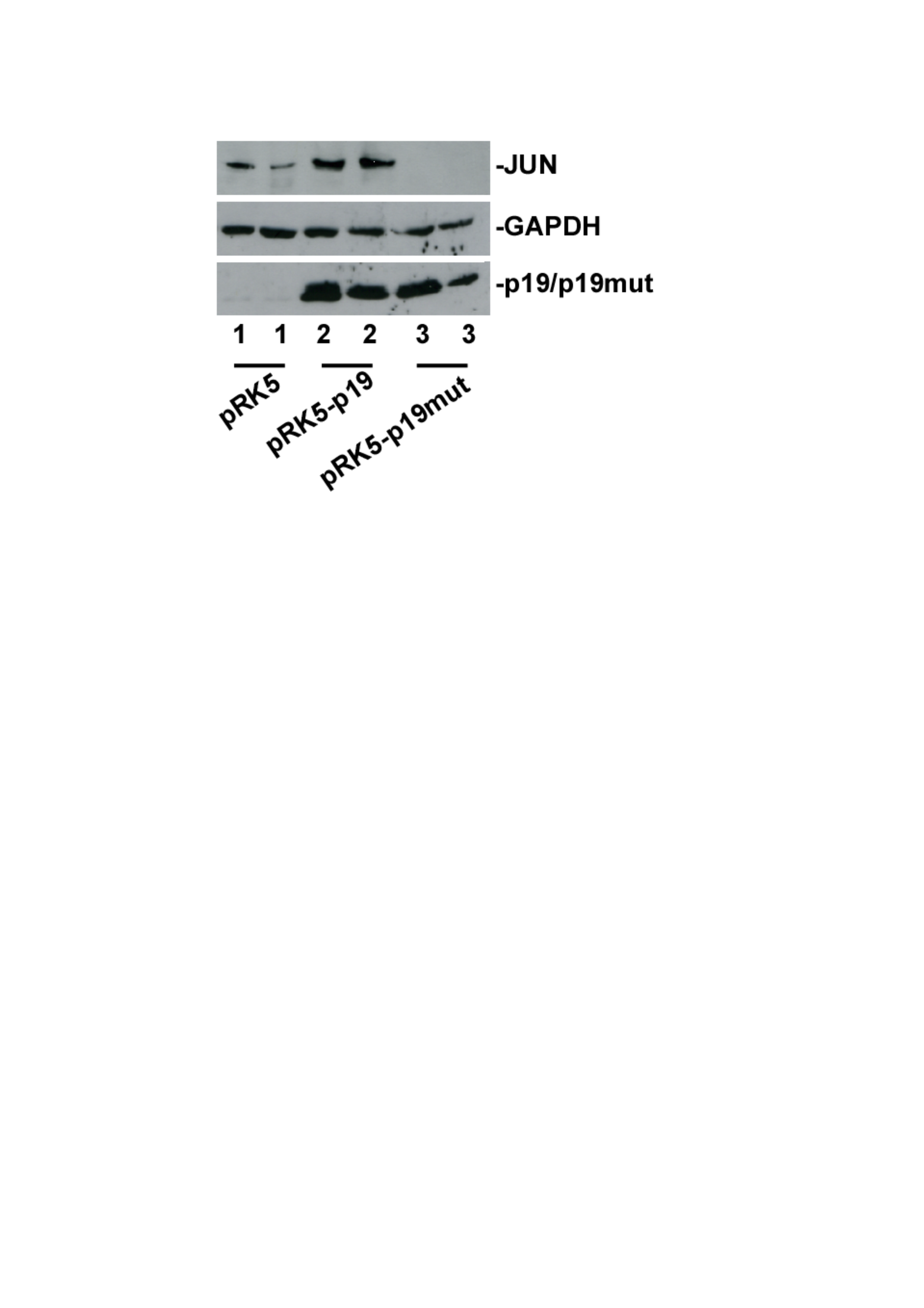

Supplement: Figure S4 — Western blot analysis of total JUN protein levels in two independent experiments HeLa cells overexpressing empty vector (lane 1), p19 (lane 2), and p19mut (lane 3). Anti-JUN, anti-GAPDH (internal control), and anti-p19 (detecting both wild-type and mutant p19) were the selected antibodies. (0.51 MB TIF) [file pone.0008513.s005.tif]

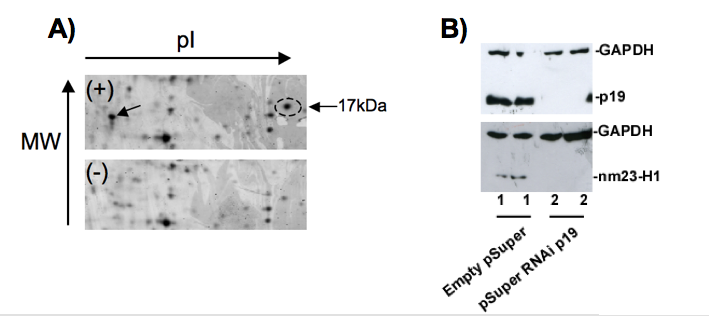

Supplement: Figure S5 — A) 2-D stained gel highlighting two sequence spots, as example of three independent experiments. (−) Negative control, proteins from HeLa cells transfected with empty vector; (+) proteins from HeLa cells overexpressing p19. Oval circle marks nm23-H1 (pI 5.8 and 17 kDa) and arrow shows overexpressed p19 (pI 4.8 and 19 kDa), which was also confirmed by microsequencing. Western blots from the same samples did not show a clear overexpression of nm23-H1; however RNAi of p19 (showed in B) showed a clear decrease on the nm23-H1 protein level, indicating that p19 regulates nm23-H1. (0.11 MB TIF) [file pone.0008513.s006.tif]
