# Supplementary material for: P19 H-Ras Induces G1/S Phase Delay Maintaining Cells in a Reversible Quiescence State
Source: PLoS One. 2009 Dec 30;4(12):e8513. doi: 10.1371/journal.pone.0008513 (PMC2798614; doi:10.1371/journal.pone.0008513)
Supplement: Table S1 — Two upper tables: Log2 is the log2 value of the fold change measuring overexpression of pRK5-p19 as compared first to pRK5 empty vector and then compared to overexpression of pRK5-p19mut in the collection of ESTs microarrays. Two lower tables: Log2 is the log2 value of the fold change measuring overexpression of pRK5-p19mut as compared first to pRK5 empty vector and then compared to overexpression of pRK5-p19. RT indicates confirmation by Real-time PCR and WB confirmation by Western blot (** P<0.001). (0.13 MB DOC) [file pone.0008513.s007.doc]

**Genes upregulated by p19 overexpression when compared to p19mut overexpression**

**Table S1**

| **Gene Name** | **Accession**  **Number** | **Homo Sapiens Genes*** | ***Log_2_*** | ***Log_2_*** | **Fold Change (**)** | **Fold**  **Change (**)** | **Fold Change** |
| --- | --- | --- | --- | --- | --- | --- | --- |
|  | | | **p19** | **p19mut** | **p19** | **p19mut** | **(p19)- (p19mut)** |
| KIR2DS1 | NM_014512 | killer cell immunoglobulin-like receptor | 2.63 | 0.38 | 6.2 | 1.3 | **4.8** |
| SLIT3 | NM_003062 | slit homolog 3 (Drosophila) | 2.29 | 0.80 | 4.9 | 1.7 | **2.8 (RT)** |
| PCSK6/PACE4 | NM_002570 | proprotein convertase subtilisin/kexin type 6 | 2.05 | 1.10 | 4.1 | 2.1 | **2.0** |
| TRIM66/TIF1D | AB002296 | mRNA KIAA0298 protein | 1.60 | 0.50 | 3.0 | 1.4 | **2.2** |
| HES4 | NM_021170 | hairy and enhancer of split 4 (Drosophila) | 0.98 | 0.24 | 2.0 | 1.2 | **1.7** |

**Genes downregulated when p19 is overexpressed versus p19mut overexpression**

| **Gene Name** | **Accession**  **Number** | **Homo Sapiens Genes*** | ***Log_2_*** | ***Log_2_*** | **Fold Change**  **(**)** | **Fold**  **Change**  **(**)** | **Fold Change** |
| --- | --- | --- | --- | --- | --- | --- | --- |
|  | | | **p19** | **p19mut** | **p19** | **p19mut** | **(p19)- (p19mut)** |
| CTSZ/CTSX | NM_001336 | cathepsin Z | -1.23 | 0.56 | -2.3 | 1.5 | **-3.5** |
| IFI27 | NM_005532 | interferon, alpha-inducible protein 27 | -0.97 | -0.43 | -2 | -1.34 | **-1.5** |
| RPRM | NM_019845 | reprimo, TP53 dependant G2 arrest mediator candidate | -0.71 | 0.15 | -1.6 | 1.1 | **-1.8** |
| SRC | NM_005417 | v-src sarcoma (Schmidt-Ruppin A-2) viral oncogene homolog (avian) | -0.65 | 0.09 | -1.6 | 1 | **-1.7(WB)** |
| RAB3GAP1 | NM_012233 | RAB3 GTPase-activating protein | -0.64 | 0.02 | -1.5 | 1.02 | **-1.6** |

**Genes upregulated when p19mut is overexpressed versus p19 overexpression**

| **Gene Name** | **Accession**  **Number** | **Homo Sapiens Genes*** | ***Log_2_*** | ***Log_2_*** | **Fold Change**  **(**)** | **Fold**  **Change**  **(**)** | **Fold Change** |
| --- | --- | --- | --- | --- | --- | --- | --- |
|  | | | **p19** | **p19mut** | **p19** | **p19mut** | **(p19mut)- (p19)** |
| MARVELD2/  TRIC | NM_144724 | MARVEL domain containing 2 | 0.53 | 1.2 | 1.4 | 2.3 | **1.6** |

**Genes downregulated when p19mut is overexpressed versus p19 overexpression**

| **Gene Name** | **Accession**  **Number** | **Homo Sapiens Genes*** | ***Log_2_*** | ***Log_2_*** | **Fold Change**  **(**)** | **Fold**  **Change**  **(**)** | **Fold Change** |
| --- | --- | --- | --- | --- | --- | --- | --- |
|  | | | **p19** | **p19mut** | **p19** | **p19mut** | **(p19)- (p19mut)** |
| ATF3 | NM_004024 | activating transcription factor 3 | 0.14 | -1.24 | 1.1 | -2.4 | **-2.6(RT)** |
| HIST2H2AA | NM_003516 | histone 2, H2aa | 0.47 | -1.21 | 1.4 | -2.3 | **-3.2** |
| TRIB3/SINK | NM_021158 | tribbles homolog 3 (Drosophila) | -0.47 | -1.1 | -1.4 | -2.1 | **-1.5** |
| JUN | NM_002228 | v-jun sarcoma virus 17 oncogene homolog (avian) | 0.49 | -1 | 1.4 | -2 | **-2.8(WB)** |
| IFIT2 | NM_001547 | interferon-induced protein with tetratricopeptide repeats 2 | -0.01 | -1 | -1 | -2 | **-2** |
| HIST1H2AD | NM_021065 | histone 1, H2ad | 0.35 | -0.9 | 1.3 | -1.9 | **-2.4** |
| HIST1H2AE | NM_021052 | histone 1, H2ae | 0.36 | -0.9 | 1.3 | -1.9 | **-2.3** |
| TXNIP/VDUP1 | NM_006472 | thioredoxin interacting protein | 0.01 | -0.82 | 1 | -1.8 | **-1.8** |
| HIST1H4H | NM_003543 | histone 1, H4h | 0.62 | -0.73 | 1.53 | -1.7 | **-2.53** |
