# Supplementary material for: P19 H-Ras Induces G1/S Phase Delay Maintaining Cells in a Reversible Quiescence State
Source: PLoS One. 2009 Dec 30;4(12):e8513. doi: 10.1371/journal.pone.0008513 (PMC2798614; doi:10.1371/journal.pone.0008513)
Supplement: Table S2 — * Putative effect of the p19 H-Ras on cancer progression. ↑ increasing of mRNA expression ↓ decreasing of mRNA expression (0.08 MB DOC) [file pone.0008513.s008.doc]

| GENE | **Mechanisms Regulated by p19** | **EFFECT ANTI OR PRO*** |
| --- | --- | --- |
| *(🡩) KIR2DS1* | To Regulate the response to viruses in natural killer cells; protective effect against Hodgkin's lymphoma | ANTI-NEOPLASIA  PHENOTYPE |
| *(🡩) SLIT3* | Diaphragm development and kidney formation during embryogenesis; cell motility and anti-metastasic target. SLIT3 RNAi reduced cell motility and Rac/Cdc42 activation | PRO-METASTASIS |
| *(🡩) PCSK6* | To contribute to the proteolitic activation of transforming growth factor (TGF)β familiy proteins. Increasing tumor cell invasiveness/metastasis; having a role in converting non-invasive keratinocytes into malignant cells | PRO-METASTASIS |
| *(🡩) TRIM66* | -- | -- |
| *(🡩) HES4* | Transcriptional repressors activated by the Notch signaling pathway. Targets of regenerative medicine and oncology. Implication of p73 in Notch pathway. Activation of Notch-1 signaling maintains the neoplastic phenotype in human Ras-transformed cells | PRO- NEOPLASIA  PHENOTYPE |
|  |  |  |
| *(🡫) CTSZ* | To regulate a cysteine proteinase Z. Increased expression and proteolytic activity of lysosomal proteases have often been positively correlated with poor prognosis for patients with a variety of malignancies, including mammary adenocarcinomas | ANTI- NEOPLASIA  PHENOTYPE |
| *(🡫) IFI27* | IFI27 is an interferon inducible protein, suggested to be a novel marker of epithelial proliferation and cancer. The increase may indicate more cell proliferation. | ANTI- NEOPLASIA  PHENOTYPE |
| *(🡫) RPRM* | Reprimo protein dependent on the p53 activity. It is induced by p53 and contributes to the cells G2 arrest. | PRO- NEOPLASIA  PHENOTYPE |
| *(🡫) SRC* | RACK1 binding protein; c-Src is a protein tyrosine kinases important in the regulation of growth and differentiation of eukaryotic cells and metastasis. There is a significant relationship between *c-SRC* activation and cancer progression. RACK1 is an inhibitor of the c-Src activity. | ANTI-METASTASIS |

| *(🡫)RAB3GAP1* | RAB3GAP1 is the the catalytic subunit of RAB3GAP and is a member of the Ras familiy of small G proteins. It is a key regulator of calcium-mediated hormone and neurotransmitter exocytosis.). Its mutations have been found in Warburg micro syndrome. | ----- |
| --- | --- | --- |

| **GENE** | **Mechanisms mis-regulated (*⌧ )* by p19mut** |
| --- | --- |
| *⌧ (🡩) MARVELD2*  */TRIC* | Membrane apposition events, such as tight junction formation and vesicular trafficking, and a tight-junction protein necessary for hearing. |
| *⌧ (🡫)*  *ATF3* | Activating transcription factor related to AP-1 complex. ATF3 knockdown significantly suppresses proliferation of wild-type cells |
| *⌧ (🡫)*  *HIST2HAA* | Histone gene in locus 1q21. Chromatin structure. |
| *⌧* *(🡫) TRIB3/SINK* | Regulatory protein for ATF4, p65/RelA and Akt/PKB. TRIB3 inhibits Akt activation by preventing its phosphorylation. RNAi-mediated knockdown of TRB3 in HepG2 cells potentiated Akt phosphorylation in response to growth factor signaling. ATF4 reagulates AP-1 transcription complex. |
| *⌧ (🡫)*  *JUN* | Transcription factor regulated by JNK. JNK binds RACK1 and this latter regulates JNK and Jun activity. Growth, differentiation, survival and apoptosis regulation. Component of the activator protein-1 (AP-1) transcription factor complex, which includes members of the FOS, MAT and ATF family. |
| *⌧ (🡫)*  *IFIT2/IFI54* | Effects on antiviral state, inhibition of cell growth and modulation of the immune system. |
| *⌧ (🡫)*  *HIST1H2AD, HIST1H2AE and HIST1H4H* | Histone gene in locus 6p21.3. Chromatin structure. |
| *⌧ (🡫)*  *TXNIP/VDUP1* | Interacts with thioredoxin that regulated AP-1 transcription complex. Overexpression of Txnip suppresses tumor growth (24) and the formation of metastases (17). RNAi of Txnip inhibits activation of p38 and JNK. Anticancer and antiproliferative agents dramatically induce Txnip expression in cancer cells |
